# Supporting Management of Noncommunicable Diseases With Mobile Health (mHealth) Apps: Experimental Study

**DOI:** 10.2196/28697

**Published:** 2022-03-02

**Authors:** Neta Kela, Eleanor Eytam, Adi Katz

**Affiliations:** 1 Shamoon College of Engineering Ashdod Israel

**Keywords:** mHealth, digital health, instrumentality, aesthetics, symbolic value, preference

## Abstract

**Background:**

Noncommunicable diseases (NCDs) are the leading global health problem in this century and are the principal causes of death and health care spending worldwide. Mobile health (mHealth) apps can help manage and prevent NCDs if people are willing to use them as supportive tools. Still, many people are reluctant to adopt these technologies. Implementing new apps could result in earlier intervention for many health conditions, preventing more serious complications.

**Objective:**

This research project aimed to test the factors that facilitate the adoption of mHealth apps by users with NCDs. We focused on determining, first, what user interface (UI) qualities and complexity levels appeal to users in evaluating mHealth apps. We also wanted to determine whether people prefer that the data collected by an mHealth app be analyzed using a physician or an artificial intelligence (AI) algorithm. The contribution of this work is both theoretical and practical. We examined users’ considerations when adopting mHealth apps that promote healthy lifestyles and helped them manage their NCDs. Our results can also help direct mHealth app UI designers to focus on the most appealing aspects of our findings.

**Methods:**

A total of 347 respondents volunteered to rate 3 models of mHealth apps based on 16 items that measured instrumentality, aesthetics, and symbolism. Respondents rated each model after reading 1 of 2 different scenarios. In one scenario, a physician analyzed the data, whereas, in the other, the data were analyzed by an AI algorithm. These scenarios tested the degree of trust people placed in AI algorithms versus the “human touch” of a human physician regarding analyzing data collected by an mHealth app.

**Results:**

As shown by the responses, the involvement of a human physician in the application had a significant effect (*P*<.001) on the perceived instrumentality of the simple model. The complex model with more controls was rated significantly more aesthetic when associated with a physician performing data analysis rather than an AI algorithm (*P*=.03).

**Conclusions:**

Generally, when participants found a human touch in the mHealth app (connection to a human physician who they assumed would analyze their data), they judged the app more favorably. Simple models were evaluated more positively than complex ones, and aesthetics and symbolism were salient predictors of preference. These trends suggest that designers and developers of mHealth apps should keep the designs simple and pay special attention to aesthetics and symbolic value.

## Introduction

### Background

Chronic diseases, known as noncommunicable diseases (NCDs), are the leading global health problem of this century [1]. According to the World Health Organization, these include cardiovascular diseases, cancer, chronic respiratory diseases, and diabetes mellitus [2]. These diseases are the principal causes of death and health care spending worldwide and are significant causes of poverty, which hinders economic development [3]. Ampofo and Boateng [4] suggested that, by 2030, the prevalence of obesity and diabetes will reach a peak in many countries. In addition, 20 million Americans are expected to have a history of cancer by 2026, an increase that coincides with the increasing prevalence of obesity [5-7].

Fortunately, many chronic diseases can be delayed until significantly later in life, or even totally prevented, if people adopt a healthy lifestyle [1]. The digital health revolution—advances in medical information technologies such as information storage, data analysis, and genetic information, together with sensors embedded in smartphones [8]—can help people maintain healthy routines and manage chronic ailments. Mobile health (mHealth) apps are software applications developed for use on small wireless computing devices such as smartphones and tablets [9,10]. These apps can potentially impact people’s health conditions because most of the global population has access to a mobile cellular network [1], and most people who have that access frequently check their phones [11]. This very high engagement level with smartphones presents an opportunity for health-oriented mHealth apps to help people lead healthier lifestyles and manage NCDs. Due to the socially authoritative influence of such apps, results can be highly effective. Still, people are often reluctant to adopt these supportive technologies, especially when they are asymptomatic [12], even though delayed treatment and intervention may, in turn, cause the disease to become irreversible.

mHealth apps serve a wide range of functions, and when adopted, they can help users cope and manage NCDs. It is estimated that such apps could cut annual US health care costs by US $150 billion by 2026 [13]. Still, the focus of most research to date has been on the judgments of physicians (eg, [1,14,15]) in postadoptive evaluations (eg, [16]). Less attention has been given to the patient’s perspective and willingness to adopt technology in pre-adoptive evaluations. Thus, this research project aimed to employ pre-use evaluations to explore factors that facilitate the adoption of mHealth apps by users who cope with NCDs.

The user interface (UI) is the first point of contact between a user and an application. Preference assigned to mHealth apps largely depends on the qualities of the UI (eg, [17,18]). Users are interested in how useful an app may be, its easy operation, and its aesthetics [18,19]. In addition, technology in health care often relies on artificial intelligence (AI). Large and complex data sets (ie, big data) are used to train algorithms to learn and improve their analysis to support decision-making [20,21]. There is great optimism that AI can substantially improve diagnostics, treatment, and support in managing NCDs [13]. Even so, while clinicians are often reluctant to trust AI [14], little research has related to the willingness of users to rely on it in managing their health. Thus, this study revolved around user perspectives in adopting mHealth apps. The study addressed 2 primary concerns. First, what UI qualities and level of complexity of mHealth apps appeal to users? Second, do users prefer their data to be analyzed by an actual physician or an AI algorithm? The contribution of this work lies within its focus on users’ considerations when adopting mHealth apps that can help them manage their NCDs.

We organize this work as follows: First, we explore the theoretical background of using technology in health care. Then, we focus on the contribution of mHealth apps in managing NCDs, followed by addressing human-computer interaction issues. Next, we propose 2 dimensions that influence preference: the quality of the design and the method by which data are analyzed (human versus AI analysis). We then continue and describe the study’s methodology and report the study results. Afterward, we discuss directions for future research, and finally, we state our conclusions.

### Technology in the Service of Health

Building on the role computers have come to play as counselors and experts, technology can promote a healthy lifestyle [22]. People assume that these “authorities” are intelligent and expect them to make suggestions and provide helpful information. Apps for healthier living that emphasize behavioral change and self-management have a high potential to help users achieve necessary lifestyle changes [23,24]. Two prominent examples of the effect of technology on health care are the Health Machine [23], which implements persuasion techniques to counter obesity and diabetes, and the Personal Aerobics Trainer, a virtual fitness trainer [25]. Technology can also support physicians in their daily tasks. For example, telemedicine enables physicians to diagnose and treat patients from afar. This technique dramatically reduces health care costs while creating a comfortable and safe treatment milieu [26]. Increased computer processing speed, the availability of large data sets, and a pool of talented AI developers have enabled the rapid development of technology in health care [27]. Moreover, AI algorithms often perform better than humans in an assortment of tasks [13].

### mHealth Apps Supporting NCDs

Because smartphones are ideally suited for collecting medical data through features such as their camera, microphone, touch-sensitive screen, and accelerometer, the use of mHealth apps is increasing exponentially throughout the world [28]. This simple and socially acceptable means of collecting behavioral and physiological information [29] can support various health conditions [30]. mHealth apps can help monitor health indicators (eg, heart rate, blood pressure). They can also support people (eg, patients with diabetes or dementia) by monitoring their illnesses (eg, chronic obstructive pulmonary disease, hypertension, diabetes, dementia) [29,31] and their caregivers (eg, physicians, nurses). They accomplish this by providing education, synchronizing records, monitoring medications, or providing access to patient information (see [32] for an extensive overview of various categories of mHealth apps). These apps can serve both general and specific purposes. Although some focus on particular health dimensions (eg, diet and physical activity), others enable personal management of well-being by monitoring a diverse range of daily behaviors with broad health-related consequences. Apps can also be managed by operating on a “manual-automatic” scale, whose extreme ends are manually activated by users on one side and fully automatic on the other [8]. Many mobile, location-based exercise apps harness the power of gamification principles on GPS-enabled smartphones [33,34]. In some of these, augmented reality turns the real world into a “game map” or playground where users play while keeping fit [35,36].

### Role of the Physician in Digital Health

Even though recent surveys indicate that more than one-third of American doctors recommend that their patients use a health or medical app [37], health care has been slow to incorporate technological advancements in clinical practice [38]. The most prominent reason for this is that the physician’s role is undergoing tremendous change [39]. Since the earliest days of professional health care, doctors represented authority and knowledge and maintained responsibility for patient treatment. This traditional role is now shifting as patients can look up their symptoms on the internet and review the opinions of others regarding the best approach for treatment. Thus, the doctor no longer is the sole medical decision maker but becomes a vital member of a health care team [39,40].

Still, although the physician’s role is changing, trust remains an essential and fundamental aspect of medical treatment [41]. In many cases, trust in the physician often plays a substantial part in patient recovery. A caring and competent physician increases this trust [40]. Perhaps this is why people turn to their doctor even though AI can provide many benefits [42]. Physicians still play a crucial role in guiding patients and helping them understand the information they encounter [40]. Because there is little regulation of medical apps or information on the internet, patients need this guidance from the doctor. In these situations, mHealth apps could be more beneficial if, before direct access to a doctor, trained AI bots can qualify whether specific symptoms warrant an actual visit [13], provided, of course, that people are willing to use them.

### Persuasive Technologies

Persuasive technologies are interactive systems designed to foster behavioral and attitudinal changes [35]. mHealth apps are technologies in which persuasive design could be beneficial, motivating people toward healthy behaviors [42-44]. According to the Fogg Behavioral Model, one of the 3 motivators for a behavior to change is social acceptance. Most people are highly motivated to do things to further this acceptance. Marcus [23] suggested that social interaction has an important impact on behavioral change. For example, people on Facebook are significantly driven by a desire for social acceptance, which is why they share pictures, beliefs, and experiences. Given that people manage their image on social media platforms, how others perceive them also seems relevant to their health habits.

Further, just as social networking sites offer platforms to share accomplishments and foster collaboration and social support [45], it is reasonable to expect that mHealth apps could likewise provide a platform for collaborating, sharing, and receiving support in the area of health activities. Indeed, one previous study showed that creating a mobile virtual community for overweight individuals allowed them to receive social support, advice, and emotional encouragement [46]. This importance of social presence and symbolism aligns with Maslow’s well-known theory of human motivation and needs [47]. The fundamental needs for *belonging and love* can be satisfied by health apps through being able to share health-related experiences with friends and family members, receive their support, and socially communicate a healthy image. In addition, the need for *esteem* can be met by displaying health-related accomplishments (such as weight and step count).

### Factors Affecting the Intention to Use mHealth Apps

Salgado et al [48] recently reviewed various studies about mobile technology solutions to address health care challenges. Following their review, they suggested that the presence of a chronic health condition predicates an impact on the acceptance of mHealth technology. Huong and Long [49] showed that the intention to use mHealth apps is affected mainly by mHealth literacy, perceived usefulness, and perceived ease of use. The concept of mHealth literacy is drawn from the compatibility suggested in Rogers’ classical theory of innovation diffusion [50]. Compatibility refers to the level at which a product is compatible with a potential user’s past experiences and beliefs. Both compatibility and literacy suggest that the more technologically literate users are, the more likely they will find innovation compatible with their values and beliefs.

Because not all users have the same level of technological literacy, the app itself should appeal to users. *Perceived usefulness* and *perceived ease of use* are constructs of the technology acceptance model, a well-known model for understanding the intention of utilizing innovative technology [51]. Perceived usefulness refers to the degree to which individuals believe that using a specific technology can improve their task performance. Perceived ease of use is the subjective belief that the product, when used, does not require significant physical or mental effort. The higher these two constructs are rated, the greater the intention to use the product [46,48]. Paying attention to these constructs in an app’s design can encourage use of these technological tools.

### Qualities of User Interfaces and Effect on Preference

Three major product qualities are essential in evaluating an interactive product: instrumentality, aesthetics, and symbolism [52]. Instrumentality relates to how a product fulfills the practical needs of promoting the users’ goals through usability. Instrumentality is an aggregate of perceived usefulness and perceived ease of use [53]. Aesthetics revolve around the sensual effect the product has on the user, eliciting an emotional reaction of, for example, tranquility, confidence, pleasantness, or frustration. Symbolism refers to the associations that the product produces and the meanings it communicates, regardless of its pragmatic goals. The effect of each of these qualities on product preference is mediated by the role of the users [18], their personal characteristics [19], and the product itself [17]. Eytam et al [18] found that the visual simplicity or complexity of a UI, as reflected by the number of its controls, influences judgments of instrumentality and aesthetics. Still, aesthetics is a consistent predictor of preference of UIs for both simple and complex designs. Symbolism is found to influence decisions about a product's characteristics. It is a salient predictor of the perceived creativity of product UIs, regardless of their complexity level [17].

### Research Hypotheses

Our model postulates that 2 significant elements influence preferences for mHealth apps. The first of these includes product qualities, namely, instrumentality, aesthetics, and symbolism [52], as reflected by the number of controls in the UI (needed to operate it) [18]. The second focuses on whether a physician or an AI algorithm performs the data analysis of information collected by the app. The following hypotheses explain how these 2 dimensions may affect preferences for different models of an mHealth app.

Because users are more likely to trust a human physician [40,41], we expect an app with a physician intervention would be rated higher in instrumental value than the same application backed by the support of an AI algorithm. Similarly, because apps presenting an excessive number of controls are reported in the literature to be more complicated to use [53,54], we expected that the number of controls will affect instrumentality ratings. Having fewer controls was expected to increase instrumental value regardless of data analysis mode [18]. Thus, H1a was that “Instrumentality judgments of mHealth apps should be higher when data are analyzed by a human physician versus an AI algorithm.” H1b was that “Instrumentality judgments of mHealth apps should be higher when there are few versus many controls, regardless of mHealth data analysis mode.”

Because the data analysis process is embedded in the system and is not reflected in the design, the presence of a human physician or an AI algorithm to analyze the data was not expected to affect noninstrumental judgments [17] that revolve around user delight and satisfaction [55]. Therefore, H2a was that “Aesthetic judgments of mHealth apps should be similar when data are analyzed either by a human physician or an AI algorithm.” H2b was that “Symbolism judgments of mHealth apps should be similar when data are analyzed either by a human physician or an AI algorithm.”

Data analysis is a pragmatic characteristic of the application that is not reflected in the design [17]. Therefore, we expected instrumentality to be a salient predictor of product preference for applications backed by a human physician and believed to have greater instrumental value. Because noninstrumental attributes are reported in the literature as salient predictors of preference [18,19], we expected aesthetics and symbolism in both application types (with a human physician and with an AI algorithm) to be salient predictors of mHealth apps*.* Therefore, H3a was that “Instrumentality should be a salient predictor of preference variance for apps that engage a human physician versus an AI algorithm.” H3b was that “Aesthetics should be a salient predictor of preference variance for apps that engage a human physician and those that engage an AI algorithm.” H3c was that “Symbolism should be a salient predictor of preference variance for apps that engage a human physician and those that engage an AI algorithm.”

Because traditional health care is characterized by personal contact (human touch) between a patient and caregiver [14,40], we expected that preferences for apps that engage a human physician would be higher than those that rely on automatic AI analysis. H4 was that “Preference is higher for apps that engage a human physician versus an AI algorithm.”

## Methods

### User Evaluation

In this research project, we conducted a user evaluation of 3 key UI features of mHealth apps: instrumentality, aesthetics, and symbolism. We compared user responses to descriptions of apps that use AI to analyze the data collected, while the app also had a physician available to analyze the same data remotely. To test if there was a difference in user preferences, we asked the 2 respondent groups to respond in writing to a questionnaire used to rate 3 different models of an mHealth app. These models differed in the number of their controls. Although each group of respondents evaluated the same models, before each group began to complete the same questionnaire, the members of one group received a different scenario than the members of the other group. The first group was told that a physician would examine the data received from the mHealth app (hereafter referred to as the physician, or doctor, scenario). The second group was told that data received by the mHealth app would be analyzed by a very accurate AI algorithm (hereafter referred to as the robot, or bot, scenario). Thus, the research was designed as a between-dimensions (2 scenarios/app descriptions) and a within-dimensions (3 models/stimuli) experiment. 

### Sample

There were 347 respondents who took part in the study (mean age 29.12, SD 9.20, range 15-86 years; gender: 198/347, 57.1% female). Respondents were volunteers recruited by students taking a data analysis course at an engineering college.

### Stimuli

The mHealth app features were designed by students participating in a UI course. The features the students were asked to create had to fit 1 of 3 themes: frequently used mHealth features, health indicators, and social-oriented features. The final designs were refined by 3 judges (2 human-computer interaction specialists and 1 biologist). The final stimuli involved 3 models: The first model was simple—with a 4-control design including frequently used mHealth features. The second model was medium—with an 8-control design presenting added health indicators. The third model was complex—with a 12-control design that included added social-oriented features (Multimedia Appendix 1 presents the 3 models of the application). Each control represented a different feature commonly used in well-being (eg, an iPhone health app) and diet-supporting applications.

### Measures

We borrowed 16 items measuring instrumentality, aesthetics, and symbolism (Multimedia Appendix 2) from the human-computer interaction literature [17].

### Manipulation

In order to manipulate the use scenario (doctor versus robot), a short introduction preceded the questionnaire and introduced either the human doctor or an automatic AI algorithm (See Multimedia Appendix 3 for each introduction).

### Procedure

Respondents were randomly assigned to 1 of 2 groups: a group presented with a doctor scenario (n=159) and a group presented with a robot scenario (n=188). The members of each group read the scenario preface for their group only, before anonymously completing the questionnaire. The illustration of each model was presented 4 times, each time with a different set of 4 randomly chosen items, to control for possible consistency effects. To conclude the study, respondents were asked to rate their preference regarding each design on a Likert scale (1-7) and choose their favorite application design.

### Ethics Approval

The Shamoon College of Engineering IRB (ethics committee) approved the research project (review 12), including the experimental task, the testing procedure, and the collection of data.

## Results

An analysis of standard residuals was carried out on the data to identify any outliers. The analysis results indicated that 22 (6.3%) of the total sample (347 respondents) needed to be removed because they responded similarly to all different items for the 3 designs tested. Of the respondents, before rating the different designs, 144 read the doctor-scenario description, and 181 read the robot-scenario description. Responses to items describing attributes of design illustrations were subjected to exploratory factor analysis. Following Rafaeli and Vilnai-Yavetz [52], our theoretical model assumed 3 distinct factors corresponding to the product qualities of instrumentality, aesthetics, and symbolism. Accordingly, 3 factors were specified for retention. Maximum likelihood estimation and oblique rotation (direct oblimin with Kaiser normalization) were applied separately to the data for each model tested (ie, Models 1, 2, and 3). Items were loaded on 3 distinct factors for all models (Multimedia Appendix 4 presents factor loadings of items for the 3 models tested). The 3 factors explained 76% to 77% of the variance in each of the 3 analyses. The items of each attribute were averaged to create scale scores. Cronbach alpha reliabilities were calculated for the attributes of each illustration in each group. All scales had adequate reliabilities (between 0.88 and 0.93) in all conditions.

In general, the correlations between the scales for the 3 models tested were between 0.62 and 0.73, which is in line with previous studies [56-58]. For Models 1 and 2, the correlations were not excessive in any of the conditions, an outcome that indicates reasonable discriminability for all 3 attribute ratings that occurred. For Model 3, the correlations between attribute ratings exceeded 0.70. These correlations may indicate that it was too difficult to differentiate between the different qualities with too many controls. Table 1 presents the correlations and reliabilities for each scale in each product condition.

**Table 1:** Correlations and reliabilities for each scale in each condition (n=325).

Level	Model 1				Model 2				Model 3		
	Instrumentality	Aesthetics	Symbolism	Instrumentality		Aesthetics	Symbolism	Instrumentality		Aesthetics	Symbolism
Instrumentality	0.91^a^	0.62	0.67	0.90^a^		0.66	0.68	0.91^a^		0.73	0.71
Aesthetics	0.62	0.92^a^	0.69	0.66		0.93^a^	0.69	0.73		0.92^a^	0.70
Symbolism	0.67	0.69	0.88^a^	0.68		0.69	0.88^a^	0.71		0.70	0.90^a^

^a^Reliability.

**Figure 1:**
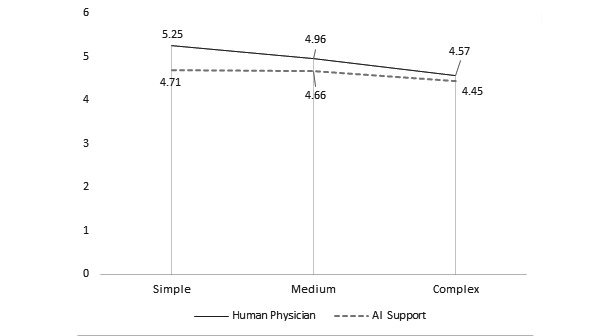
Average instrumentality ratings of doctor versus robot based on model. AI: artificial intelligence.

**Figure 2:**
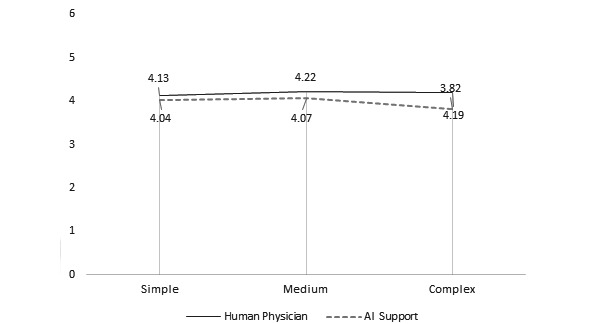
Average aesthetics ratings of doctor versus robot based on model. AI: artificial intelligence.

**Figure 3:**
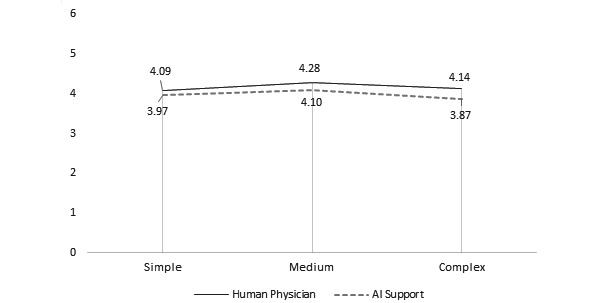
Average symbolism ratings of doctor versus robot based on model. AI: artificial intelligence.

**Figure 4:**
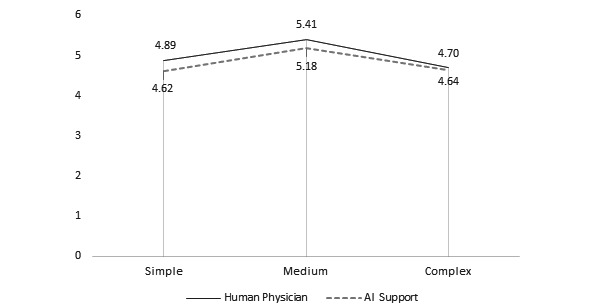
Average preference ratings of doctor versus robot based on model. AI: artificial intelligence.

A series of mixed-design analysis of variance studies were conducted with *product* (doctor versus robot) as a between-groups factor and *model* (1, 2, or 3) as a within-subjects factor. *Instrumentality, aesthetics, symbolism,* and *preference* were the dependent variables. The Mauchly test indicated that the assumption of sphericity had been violated (instrumentality: χ^2^_2_=130.28, *P*<.001; aesthetics: χ^2^_2_=51.74, *P*<.001; symbolism: χ^2^_2_=48.54, *P*<.001; preference: χ^2^_2_=75.29, *P*<.001). Therefore, the degrees of freedom were corrected using Greenhouse-Geisser estimates of sphericity (instrumentality: ε=0.76; aesthetics: ε=0.88; symbolism: ε=0.88; preference: ε=0.83). All pairwise comparisons used the Bonferroni correction for multiple tests. Figures 1-4 detail ratings for product attributes for the 2 product conditions tested.

*Model* had a significant effect on instrumentality, aesthetics, symbolism, and preference ratings. There was a positive relationship between model and instrumentality ratings. The Model 1 (doctor: mean 5.25, SD 1.57; robot: mean 4.71, SD 1.59) and Model 2 (doctor: mean 4.96, SD 1.56; robot: mean 4.66, SD 1.50) designs were rated as significantly more instrumental than that of Model 3 (doctor: mean 4.57, SD 1.72; robot: mean 4.45, SD 1.71) in both conditions (both comparisons, *P*<.001). Similar trends were also reported in studies that examined the effect of model choice on judgments of instrumentality [18,19]. There was a significant difference between aesthetic ratings of Model 2 (mean 4.07, SD 1.60) versus Model 3 (mean 3.82, SD 1.60) but only in the robot condition (*P*=.004). There was a significant difference between the symbolism ratings of Model 2 (doctor: mean 4.28, SD 1.60; robot: mean 4.10, SD 1.48) compared with those of Model 3 (doctor: mean 4.14, SD 1.65; robot: mean 3.87, SD 1.55; *P*=.001). Finally, there was a significant difference between preference ratings for Model 1 (doctor: mean 4.89, SD 1.77; robot: mean 4.62, SD 1.88) and Model 3 (doctor: mean 4.70, SD 1.8760; robot: mean 4.64, SD 1.83) compared with those of Model 2 (doctor: mean 5.41, SD 1.32; robot: mean 5.18, SD 1.36; *P*<.001).

*Group* had a significant effect on the instrumentality ratings of Model 1 (doctor: mean 5.25, SD 1.57; robot: mean 4.71, SD 1.59; *P*<.001) and on the aesthetics ratings of Model 3 (doctor: mean 4.19, SD 1.70; robot: mean 3.82, SD 1.60; *P*=.03). The interaction effect was significant for the *instrumentality* (*F*_2,646_=8.05, *P*=.001) and *aesthetics* rating (*F*_2,646_=4.19, *P*=.02), indicating that the effect of the virtual presence of a human physician was greater in judgments of instrumentality of Model 1 and in judgments of aesthetics of Model 3.

We conducted separate regression analyses for each model (1, 2, and 3), with preference as the dependent variable and product attributes (instrumentality, aesthetics, and symbolism) as the predictors. Tests to see if the data met the assumption of collinearity indicated that multicollinearity was not a concern for any of the analyses conducted (tolerance >.2) [59]. The results of the regression are presented in Table 2 and Table 3. In the doctor condition, independent variables (product attributes) accounted for 13% to 18% of the preference variance in the Models 1, 2, and 3 analyses. When reading the doctor scenario before evaluating Model 1, respondents considered all product attributes as salient. When reading the robot scenario before evaluating Model 1, respondents considered only aesthetics as a salient predictor for preference. When preparing to evaluate Model 2, respondents who read either scenario found one noninstrumental attribute as salient (although they found different attributes—symbolism for the doctor scenario and aesthetics for the robot). The 2 different scenarios did not influence the importance of any of the 3 product attributes in any significant manner. Probably adding many controls to the model, as in Model 3, brings about different considerations that we did not measure in this research.

To test the percentage of respondents preferring different designs after reading each scenario, we used a Z-ratio test (based on the calculator in [60]). The Z-ratios for proportions of design choice frequencies and group were not significant for the simple design (Z=0.455, *P=*.65), medium design (Z=–0.210*,* *P*=.48), and Model 3 (Z=–0.211, *P=*.83). The frequencies of the choice of design are depicted in Table 4.

**Table 2:** Preference model standardized regression coefficients (doctor [n=144] scenario).

Condition	Doctor model 1 (*R*^2^=0.18)					Doctor model 2 (*R*^2^=0.13)					Doctor model 3 (*R*^2^=0.17)			
	Beta	SE	*P* value	*F* (df)	Beta		SE	*P* value	*F* (df)	Beta		SE	*P* value	*F* (df)
Instrumentality	–0.34	0.13	*P*=.004	10.40 (3,140)	0.13		0.11	*P*=.34	6.87 (3,140)	0.20		0.16	*P*=.17	9.68 (3,140)
Aesthetics	0.32	0.11	*P*=.004		0.02		0.10	*P*=.88		0.14		0.14	*P*=.29	
Symbolism	0.38	0.11	*P*=.001		0.25		.09	*P*=.02		0.12		0.13	*P*=.28	

**Table 3:** Preference model standardized regression coefficients (robot [n=181] scenario).

Condition	Robot model 1 (*R*^2^=0.27)					Robot model 2 (*R*^2^=0.16)					Robot model 3 (*R*^2^=0.11)			
	Beta	SE	*P* value	*F* (df)	Beta		SE	*P* value	*F* (df)	Beta		SE	*P* value	*F* (df)
Instrumentality	–0.10	0.11	*P*=.25	21.63 (3,177)	–0.06		0.09	*P*=.55	11.38 (3,177)	0.04		0.11	*P*=.71	7.37 (3,177)
Aesthetics	0.48	0.12	*P*=.001		0.01		0.09	*P*=.95		0.13		0.13	*P*=.26	
Symbolism	0.12	0.14	*P*=.33		0.43		0.12	*P*=.001		0.20		0.14	*P*=.10	

**Table 4:** Choices of design (doctor versus robot scenario; total n=325).

Scenario	Model 1 (n=84), n	Model 2 (n=108), n	Model 3 (n=133), n
Doctor (n=144)	39	47	58
Robot (n=181)	45	61	75

## Discussion

### Principal Findings

This research project investigated user preferences for mHealth apps. We sought to facilitate the acceptability of such technology in health care provision, which would lead to more frequent and productive use of these apps. In general, when a human touch was present in the analysis, that is, when the respondents thought a physician would analyze the data collected by the mHealth app, ratings of both instrumentality and aesthetics were higher than the scenario in which they thought AI would analyze their data. These overall higher ratings can be explained by trust. Previous studies reported that people do not trust AI-based technology in health care as much as they do their doctors (eg, [15,42]). A human physician increases the sense of connectedness to a knowledgeable, caring health care professional [41].

In contrast, an AI algorithm works as a “black box”—a metaphor suggesting that, because people do not know how they produce their outputs, they have less trust in them [15]. Vo et al [61] reviewed 43 qualitative studies of patients’ perceptions of mHealth. They found that patients appreciated communicating directly with health care professionals and providers because they could receive responses to their concerns from a person who cared. Patients with chronic ailments reported that they want to share their health records with their physicians between clinic visits [62] because of their need for a relationship with the caregiver [41]. Table 5 summarizes the research hypotheses.

**Table 5:** Research hypotheses.

Hypothesis number	Hypothesis description	Model 1^a^	Model 2^b^	Model 3^c^	Table or figure
H1^a^	Instrumentality judgments of mHealth^d^ apps should be higher when data are analyzed by a human physician versus an AI^e^ algorithm.	√	X	X	Figure 1
H1^b^	Instrumentality judgments of mHealth apps should be higher when there are few versus many controls, regardless of mHealth data analysis mode.	√	√	√	Figure 1
H2^a^	Aesthetic judgments of mHealth apps should be similar when data are analyzed either by a human physician or an AI algorithm.	√	√	X	Figure 2
H2^b^	Symbolism judgments of mHealth apps should be similar when data are analyzed either by a human physician or an AI algorithm.	√	√	√	Figure 3
H3^a^	Instrumentality should be a salient predictor of preference variance for apps that engage a human physician versus an AI algorithm.	√	X	X	Table 2
H3^b^	Aesthetics should be a salient predictor of preference variance for apps that engage a human physician and those that engage an AI algorithm.	√	X	X	Table 2
H3^c^	Symbolism should be a salient predictor of preference variance for apps that engage a human physician and those that engage an AI algorithm.	X	√	X	Table 2
H4	Preference is higher for apps that engage a human physician versus an AI algorithm.	X	X	X	Figure 4

^a^4 controls in the design.

^b^8 controls in the design.

^c^12 controls in the design.

^d^mHealth: mobile health.

^e^AI: artificial intelligence.

The simplest model (Model 1 with 4 controls) was judged the most instrumental among the 3 models tested. Predictably, the most complex model (Model 3 with 12 controls) was regarded as the least instrumental. This pattern of rating simplicity as providing high instrumentality has been noted in previous research [18,19]. Usability experts often advocate simplicity to promote a product’s usability. They suggest that simple designs help people achieve their goals more efficiently and effectively because of their clarity and filtering out unnecessary features [63]. Hilliard et al [64] reported that chronically ill patients preferred apps that required minimal effort to input medical data or to set up scheduled alarms. In addition, respondents in our study, regardless of the scenario they read before responding in writing to the survey, preferred mid-level complexity (Model 2 with 8 controls). This preference for Model 2 hints at the idea that, while users do not want restricted functionality, they also do not want feature-laden apps [17,18].

The complex design was rated significantly more aesthetic when a human physician analyzed the data than an AI algorithm. Simplicity is often associated with beauty [63] and sophistication [65,66]. The effect of a human physician’s involvement on aesthetic perceptions could be derived from a halo effect that made the overall impression of the application more positive in general because of this feature. Even so, previous research found that aesthetic websites enhance customer trust [67-69]. Perhaps this effect is also reversed, and confidence in a human physician’s involvement in the app made it appear more aesthetic.

Noninstrumental qualities, namely, aesthetics and symbolism, were significant predictors of preference variance in both types of eHealth applications tested, which hints at the salient role of hedonic qualities in the evaluation of the app. Although potential users of mHealth apps have primarily utilitarian needs [55], users of technology products tend to stress hedonic motivations [56,57]. Eytam et al [18] noted that aesthetics is a consistent predictor of preference variance. The negative effect of instrumentality on product preference when a human physician is involved in data analysis may suggest that users’ needs are not settled when their usability expectations are met but rather that they seek the hedonic benefits of the app.

This study explored how mHealth app qualities can affect the willingness of patients with NCDs to adopt these tools in their daily routine. Although it included the primary app qualities of instrumentality, aesthetics, and symbolism, it did not delve into the specific functions that patients look for in mHealth apps. That said, the literature suggests that specific functions such as connectedness to a support group through social media can promote mHealth apps [23]. Future studies should relate to particular features in these apps that can encourage willingness to adopt them. Specifically, future research should examine how widening the human touch in applications via connectedness to support groups may affect the acceptability of mHealth apps.

### Conclusion

Our research model proposed 2 dimensions that influence app preference: design quality and the method of data analysis (ie, by a human physician or AI algorithm analysis). We tested 3 application models to study these factors, each with a different number of controls for the various functions. Initially, we hypothesized that human touch in the application in the form of an assumed analysis of the data by a human physician would be perceived as more attractive than one automatically analyzed by an AI algorithm. The involvement of a physician in the application had a significant effect on the perceived instrumentality only for the simple design; however, physician involvement did not affect preference for an app. This lack of ability to affect preference is probably because judgments of the noninstrumental qualities—aesthetics and symbolism—which are the significant predictors of preference variance, were unaffected by how the data were analyzed. Overall, our findings show that mHealth adoption can be facilitated when the complexity of the design is restricted, when hedonic qualities of the design are attended to, and when human touch with a physician is taken into account. Because previous research suggests that aesthetics enhance trust in technology, investing in the aesthetics of mHealth apps would be a wise strategy to promote adoption by potential users.

## Appendix

Multimedia Appendix 1 Stimuli.

Multimedia Appendix 2 Items.

Multimedia Appendix 3 Scenarios.

Multimedia Appendix 4 Factor loadings.

## Abbreviations

AI: artificial intelligence
mHealth: mobile health
NCD: noncommunicable disease
UI: user interface
