# Supplementary figures and images for: Supporting Management of Noncommunicable Diseases With Mobile Health (mHealth) Apps: Experimental Study
Source: JMIR Hum Factors. 2022 Mar 2;9(1):e28697. doi: 10.2196/28697 (PMC8928053; doi:10.2196/28697)

## Stimuli

| **Simple** | **Medium** | **Complex** |
| --- | --- | --- |
| 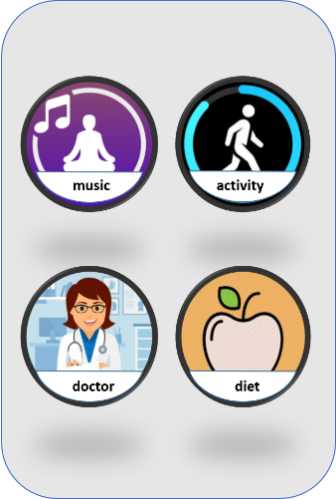 | 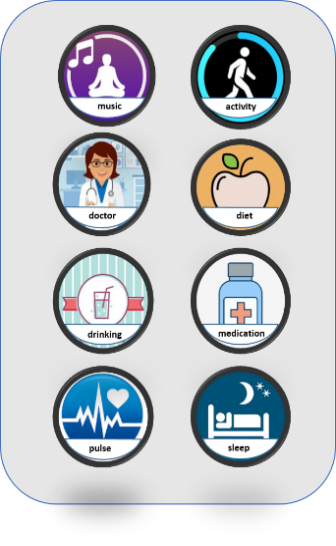 | 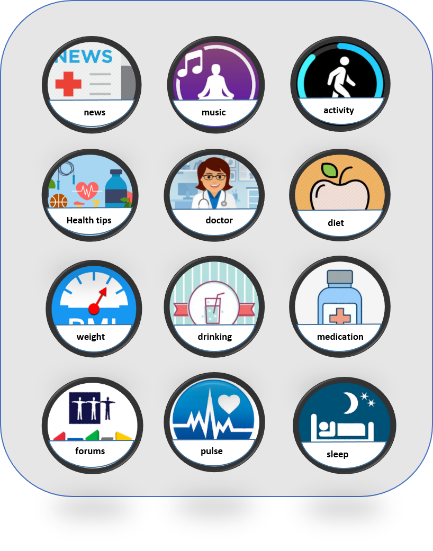 |

Supplement: Multimedia Appendix 1 [file humanfactors_v9i1e28697_app1.docx]
