# Supplementary material for: Supporting Management of Noncommunicable Diseases With Mobile Health (mHealth) Apps: Experimental Study
Source: JMIR Hum Factors. 2022 Mar 2;9(1):e28697. doi: 10.2196/28697 (PMC8928053; doi:10.2196/28697)
Supplement: Multimedia Appendix 2 [file humanfactors_v9i1e28697_app2.docx]

## Items

| **Attribute** | | **Item** |
| --- | --- | --- |
| I1 | **Instrumentality** | It is easy to learn how to use the application |
| I2 |  | It is easy to use the application |
| I3 |  | It is convenient to use the application |
| A3 | **Aesthetics** | The design is pleasurable |
| A4 |  | The design is artistic |
| A5 |  | The design is creative |
| S1 | **Symbolism** | The application represents who I am |
| S2 |  | The application fits my personality |
| S3 |  | The application fits me |
