# Supplementary material for: Supporting Management of Noncommunicable Diseases With Mobile Health (mHealth) Apps: Experimental Study
Source: JMIR Hum Factors. 2022 Mar 2;9(1):e28697. doi: 10.2196/28697 (PMC8928053; doi:10.2196/28697)
Supplement: Multimedia Appendix 3 [file humanfactors_v9i1e28697_app3.docx]

## Scenarios

A Human Physician Scenario

Your family doctor has recommended using a health app. This app allows tracking health data like weight and heart rate, lifestyle tracking like nutrition and sleep, and inspiring activities like music, health tips, and participation in health-related forums. Exceptional data are communicated to a human physician. You will be asked to rate three app models. The following pages will show you the different models with items related to their properties. Please rate how the following sentences reflect your opinion (1 - not at all reflective, 7 - definitely reflects).

A Bot Support Scenario

Your friends have recommended using a health app. This app allows tracking health data such as weight and heart rate, lifestyle tracking like nutrition and sleep, and inspiring activities such as music, health tips, and a conversation with a medical robot (bot) who can generally advise on health issues. You will be asked to rate three app models. The following pages will show you the different models with items related to their properties. Please rate how the following sentences reflect your opinion (1 - not at all reflective, 7 - definitely reflects).
