# Supplementary material for: Supporting Management of Noncommunicable Diseases With Mobile Health (mHealth) Apps: Experimental Study
Source: JMIR Hum Factors. 2022 Mar 2;9(1):e28697. doi: 10.2196/28697 (PMC8928053; doi:10.2196/28697)
Supplement: Multimedia Appendix 4 [file humanfactors_v9i1e28697_app4.docx]

## Factor Loadings

| **VSL** | **Simple** | | | **Medium** | | | **Complex** | | |
| --- | --- | --- | --- | --- | --- | --- | --- | --- | --- |
| **Item** | **1** | **2** | **3** | **1** | **2** | **3** | **1** | **2** | **3** |
| I1 |  | 0.83 |  |  | 0.86 |  | 0.84 |  |  |
| I2 |  | 0.95 |  |  | 0.86 |  | 0.89 |  |  |
| I3 |  | 0.77 |  |  | 0.64 |  | 0.70 |  |  |
| A3 | 1.00 |  |  | 0.96 |  |  |  | -0.96 |  |
| A4 | 0.73 |  |  | 0.69 |  |  |  | -0.72 |  |
| A5 | 0.76 |  |  | 0.86 |  |  |  | -0.68 |  |
| S1 |  |  | 0.83 |  |  | -0.91 |  |  | 0.87 |
| S2 |  |  | 0.58 |  |  | -0.51 |  |  | 0.65 |
| S3 |  |  | 0.87 |  |  | -0.71 |  |  | 0.81 |
